# Survival impacts of extent of resection and adjuvant radiotherapy for the modern management of high-grade meningiomas

**DOI:** 10.1007/s11060-019-03278-w

**Published:** 2019-09-06

**Authors:** Depei Li, Pingping Jiang, Shijie Xu, Cong Li, Shaoyan Xi, Ji Zhang, Yinsheng Chen, Xiaobing Jiang, Xiangheng Zhang, Ke Sai, Jian Wang, Yonggao Mou, Chao Ke, Zhongping Chen

**Affiliations:** 1 Department of Neurosurgery and Neuro-Oncology, State Key Laboratory of Oncology in South China, Collaborative Innovation Center for Cancer Medicine, Sun Yat-Sen University Cancer Center, 651 Dongfeng Road East, Guangzhou, 510060 China; 2 Department of Traditional Chinese Medicine, The First Affiliated Hospital of Guangdong Pharmaceutical University, Guangzhou, China; 3 Department of Pathology, State Key Laboratory of Oncology in South China, Collaborative Innovation Center for Cancer Medicine, Sun Yat-Sen University Cancer Center, Guangzhou, China

**Keywords:** Meningioma, Extent of resection, Radiotherapy, SEER, Propensity score matching

## Abstract

**Purpose:**

We aim to investigate the impacts of extent of resection and adjuvant radiotherapy on survival of high-grade meningiomas (WHO grade II–III) according to modern diagnosis and management.

**Methods:**

Patients with high-grade meningiomas were identified in the Surveillance Epidemiology and End Results (SEER) database between 2000 and 2015 and used for survival analysis. Propensity score matching (PSM) was conducted to reduce selection bias. Another 92 patients from Sun Yat-sen University Cancer Center (SYSUCC) were used for validation.

**Results:**

530 patients were enrolled from SEER. Patients with gross total resection (GTR) had no significantly different overall survival (OS) compared with those with subtotal resection (STR), even after performing PSM between these two groups. Multivariable analysis found that age ≥ 65 years (HR 2.22, P < 0.001), tumor diameter > 6 cm (HR 1.59, P = 0.004) and grade III tumor (HR 4.31, P < 0.001) were associated with worse OS. Stratification analysis showed that adjuvant radiotherapy conferred significantly improved OS for grade III meningiomas, but not for grade II meningiomas, regardless of resection extent. In SYSUCC cohort, resection extent was also not significantly associated with OS. However, patients with GTR (Simpson grade I–III) had distinctly increased progression-free survival (PFS) than those with STR (P < 0.001). Additionally, for grade II meningiomas after GTR, radiotherapy was unable to improve OS and PFS.

**Conclusion:**

On modern management of high-grade meningiomas, GTR does not improve OS, but seems to be associated with increased PFS. Radiotherapy is reasonable as a supplement for treating grade III meningiomas, whereas its effect for grade II meningiomas remains uncertain and needs further validation by prospective study.

**Electronic supplementary material:**

The online version of this article (doi:10.1007/s11060-019-03278-w) contains supplementary material, which is available to authorized users.

## Introduction

Meningiomas are the most common primary intracranial tumors in adults, accounting for over 35% of all primary central nervous system (CNS) tumors [1]. Meningiomas are classified into three pathological grades according to the World Health Organization (WHO) definition [2]. Although the majority of meningiomas are benign tumors (WHO grade I) and can be cured by gross total resection (GTR), about 20% are high-grade (WHO grade II and III), and show more malignant behavior [1, 2]. The higher histological grade indicates increased risk of tumor recurrence and mortality [2, 3]. Before 2000, diagnosis and classification of meningiomas as grade II or grade III were highly subjective, but these have been gradually improved by using more detailed standardizations including establish of cutoff values of mitotic counts (WHO 2000 criteria) and introduction of brain invasion (WHO 2007 criteria) for grading assessment [2, 4]. This modification caused increase of the incidence of high-grade meningiomas and showed more power to predict clinical outcomes [5, 6].

Surgical resection is the first choice to treat high-grade meningiomas and should aim to achieve GTR [7], corresponding to Simpson grade I-III resection [8]. After surgery, radiation is usually used as adjuvant therapy at many institutions for patients with grade III meningiomas (even after GTR) and incompletely resected grade II meningiomas [7]. It has been reported that GTR and adjuvant radiation were associated with improved local control of the tumor [5, 9, 10]. However, the long-term impacts of GTR and radiotherapy for patients’ survival are still unclear, even on the modern management of high-grade meningiomas according to the adapted WHO diagnostic criteria (WHO 2000/2007 editions) [2, 7].

Sponsored by the National Cancer Institute, the Surveillance Epidemiology and End Results (SEER) program collects and publishes cancer incidence, treatment and survival data from 18 population-based cancer registries, which cover more than 25% of the US population. In this study, 530 adult patients with primary intracranial high-grade meningiomas were identified from the SEER registry, and another 92 contemporaneous patients from Sun Yat-sen University Cancer Center (SYSUCC) were used as a validation cohort, to investigate the survival impacts of extent of resection and adjuvant radiotherapy on modern meningioma management.

## Methods

### Patients and data collection

Patients with a diagnosis of high-grade meningiomas between the year 2000 and 2015 were identified using the SEER*Stat software (Version 8.3.5) and “incidence-SEER 18 registries Custom Data (with additional treatment fields), Nov 2018 Sub” dataset, with International Classification of Diseases for Oncology, Third Edition (ICD-O-3) histology codes 9530–9539 (meningiomas) and behavior codes *borderline* and *malignant*. Patients were excluded if (1) not primary or first tumor; (2) intraspinal lesions; (3) not histological confirmation; (4) ICD-O-3 grade I and CS site specific WHO grade I; (5) no surgery performed; (6) receipt of pre- and intra-operative radiation. Lastly, only the patients aged 18–79 years and survival > 1 month were enrolled. The flow diagram of patient selection is depicted in supplemental Fig. S1. According to the coding manual of the SEER program (available at: seer.cancer.gov/tools/codingmanuals), patient demographics (age, race, gender and year of diagnosis), tumor characteristics (location, laterality, size, bone invasion and WHO grade), treatment records (extent of resection and radiotherapy) and overall survival (OS) status and time were collected. Of note, definition of GTR and details of resection related to Simpson grade [8] are not available from SEER. Instead, Extent of resection was characterized as GTR, subtotal resection, partial resection or excision biopsy. The vast majority of the patients received external beam radiotherapy (EBRT), except one patient received radioisotopes and another whose treatment was not specified.

 To validate the findings from the SEER cohort, 92 adult patients with high-grade meningiomas that received treatment at the SYSUCC from 2000 to 2015 were retrospectively analyzed. The pathology was reviewed and diagnosed according to the WHO 2007 classification. Simpson grading system is used for assessment of extent of resection [8]. Patients were followed-up until October 2018. OS was defined as the duration from the date of surgery to death. Progression-free survival (PFS) was defined as the duration from surgery to tumor recurrence detected by magnetic resonance imaging (MRI) or death. Informed consent was obtained in compliance with the Ethics and the Medical Institutional Review Board at Sun Yat-sen Caner Center.

## Propensity score matching (PSM)

For SEER cohort, a propensity score study was conducted to reduce bias in patient selection. Propensity scores were estimated using a logistic regression model based on both covariables including year of diagnosis, gender, age, race, tumor location, laterality, size, WHO grade, bone invasion and radiotherapy that might affect survival independent of extent of resection. One-to-one matching without replacement was employed with a caliper width of 0.05. The quality of matching was evaluated by comparison of each covariable after PSM.

### Statistical analysis

SPSS software version 20 (IBM corp., Santa Monica, CA) was used for statistical analysis. Categorical variables were compared by chi-squared test. Survival curves were plotted by Kaplan–Meier method and compared using a log-rank test. Multivariate Cox proportional hazards models were constructed using a stepwise backward method, adjusting for variables previously associated with survival in univariate analysis at P < 0.20 level. Logistic regression model was applied to assess the likelihood of receiving adjuvant radiation. All statistical tests were two-sides and statistical significance was defined as P < 0.05.

## Results

### Survival impact of GTR before and after PSM

**Fig. 1:**
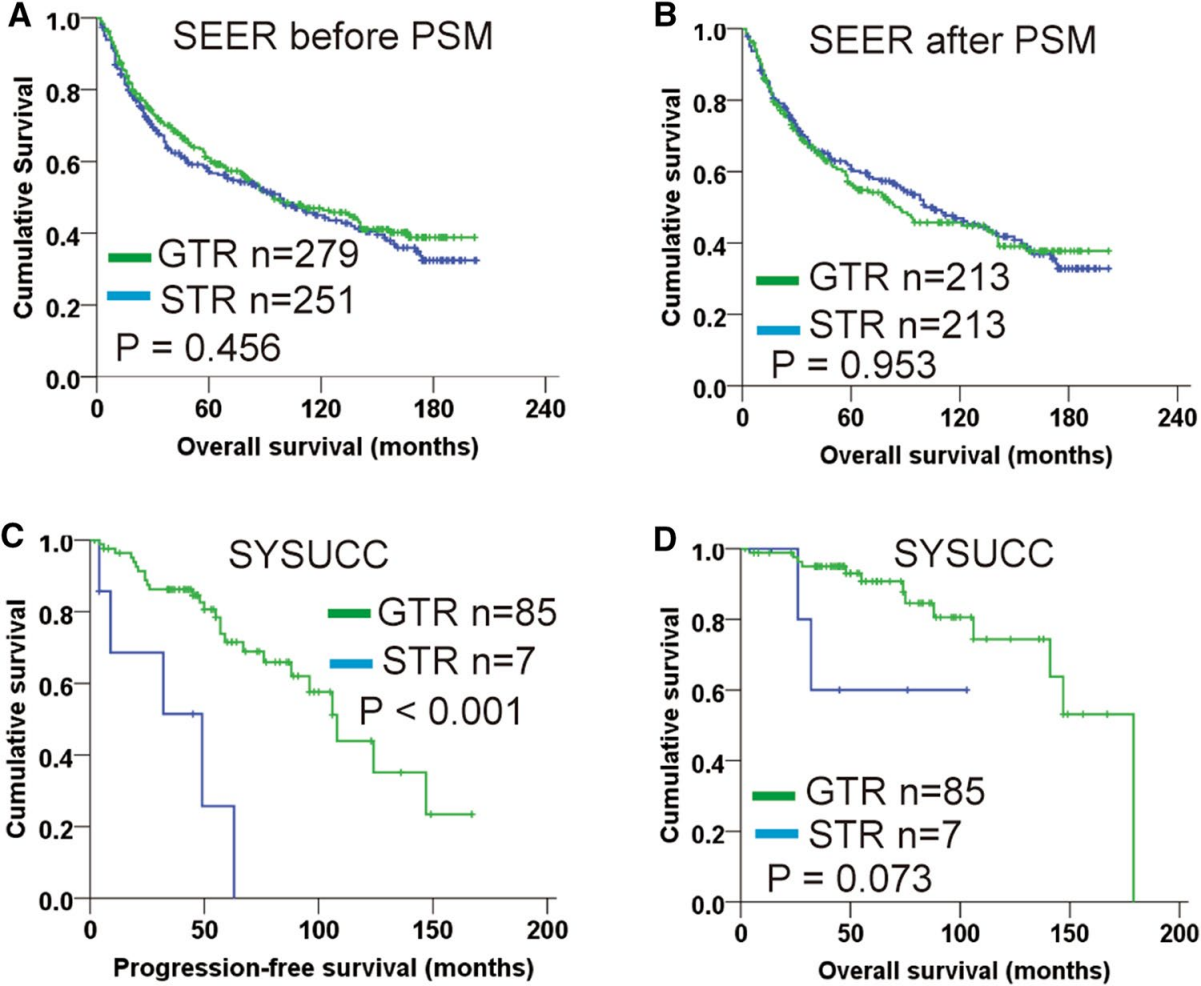
Kaplan–Meier plot for extent of resection on patients with high-grade meningiomas. **a** Overall survival analysis for SEER cohort before PSM; **b** Overall survival analysis for SEER cohort after PSM; **c** Progression-free survival analysis for SYSUCC cohort; **d** Overall survival analysis for SYSUCC cohort.* PSM* propensity score matching,* GTR* gross total resection,* STR* subtotal resection

A total of 530 adult patients with primary intracranial high-grade meningiomas were enrolled; among them, 279 achieved GTR and 233 received postoperative radiotherapy. Baseline characteristics of patients undergoing GRT or not was concluded in Table 1. The proportion of patients undergoing GTR distinctly dropped from 57.1% between 2000 and 2007 to 47.3% between 2008 and 2015 (P = 0.025). Median OS of patients received GTR and those who received subtotal or partial resection (STR) were 95.0 months (95% CI 61.8–128.2 months) and 100.0 months (95% CI 68.2–131.8 months), respectively. The survival difference was not significant (P = 0.456; Fig. 1a).

**Table 1:** Clinical characteristics of patients with high-grade meningiomas from the SEER database

Variables (n, %)	Total (n = 530)	Total resection (n = 279)	Subtotal resection (n = 251)	P value
Year of diagnosis				
2000–2007	289 (54.5)	165 (59.1)	124 (49.4)	**0.025**
2008–2015	241 (45.5)	114 (40.9)	127 (50.6)	
Gender				
Male	244 (46.0)	128 (45.9)	116 (46.2)	0.938
Female	286 (54.0)	151 (54.1)	135 (53.8)	
Age				
< 65	326 (61.5)	170 (60.9)	156 (62.2)	0.773
≥ 65	204 (38.5)	109 (39.1)	95 (37.8)	
Race				
White	389 (73.4)	204 (73.1)	185 (73.7)	0.808
Black	83 (15.7)	46 (16.5)	37 (14.7)	
Other	58 (10.9)	29 (10.4)	29 (11.6)	
Tumor location				
Supratentorial	254 (47.9)	138 (49.5)	116 (46.2)	0.555
Infratentorial	159 (30.0)	78 (27.9)	81 (32.3)	
Unknown	117 (22.1)	63 (22.6)	54 (21.5)	
Laterality				
Left	179 (33.8)	95 (34.1)	84 (33.4)	0.892
Right	158 (29.8)	80 (28.7)	78 (31.1)	
Bilateral	32 (6.0)	16 (5.7)	16 (6.4)	
Unknown	161 (30.4)	88 (31.5)	73 (29.1)	
Tumor diameter				
≤ 6 cm	289 (54.5)	166 (59.5)	123 (49.0)	**0.048**
> 6 cm	86 (16.2)	42 (15.1)	44 (17.5)	
Unknown	155 (29.2)	71 (25.4)	84 (33.5)	
Bone invasion				
Yes	141 (26.6)	71 (25.4)	70 (27.9)	0.526
No	389 (73.4)	208 (74.6)	181 (72.1)	
WHO grade				
II	70 (13.2)	38 (13.6)	32 (12.7)	0.788
III	178 (33.6)	90 (32.3)	88 (35.1)	
Unknown	282 (53.2)	151 (54.1)	131 (52.2)	
Radiotherapy				
Yes	233 (44.0)	119 (42.7)	114 (45.4)	0.522
No	297 (56.0)	160 (57.3)	137 (54.6)	

Chi-squared test was used for comparison

P < 0.05 were in bold

As the distributions of year of diagnosis and tumor size were significantly difference between GTR and STR group (P = 0.025 and P = 0.048, respectively; Table 1), PSM was conducted to minimize selection bias. 213 pairs of patients were subsequently generated and both covariables entered PSM were balanced as shown in supplemental Table S1 (all P > 0.05). After PSM, the survival difference between patients with GTR and those with STR remained not significant (P = 0.953; Fig. 1b).

### Cox proportional hazards regression analysis for the SEER cohort

The result of Cox regression for the whole SEER cohort was shown in Table 2. Year of diagnosis after 2008 (P = 0.008), male sex (P = 0.001), age ≥ 65 years (P < 0.001), tumor with a diameter > 6 cm (P < 0.001), grade III tumor (P < 0.001), bone invasion (P = 0.047) and receipt of radiotherapy (P = 0.001) were associated with worse OS. Multivariate analysis found that age at diagnosis, tumor size and WHO grade were independent survival predictors.

**Table 2:** Risk factors for overall survival on patients with high-grade meningiomas from the SEER database (n = 530)

Variables	Univariate analysis			Multivariate analysis		
	HR	95% CI	P value	HR	95% CI	P value
Year of diagnosis (2008–2015/2000–2007)	1.42	1.10–1.83	**0.008**	–		
Gender (male/female)	1.49	1.18–1.89	**0.001**	1.28	1.00–1.63	0.054
Age (≥ 65y/< 65 y)	2.33	1.84–2.95	** < 0.001**	2.22	1.75–2.82	** < 0.001**
Race						
White	1(ref)					
Black	0.98	0.70–1.36	0.881			
Other	1.25	0.86–1.82	0.242			
Tumor location						
Supratentorial	1(ref)					
Infratentorial	1.21	0.92–1.58	0.174	–		
Unknown	0.91	0.69–1.26	0.535			
Laterality						
Left	1(ref)					
Right	0.93	0.69–1.25	0.61			
Bilateral	1.20	0.72–1.98	0.49			
Unknown	0.65	0.48–0.87	0.004			
Tumor diameter						
≤ 6 cm	1(ref)			1(ref)		
> 6 cm	1.95	1.42–2.68	** < 0.001**	1.59	1.16–2.20	**0.004**
Unknown	1.40	1.07–1.82	0.015	1.40	1.06–1.84	**0.017**
WHO grade						
II	1(ref)			1(ref)		
III	4.74	2.67–8.43	** < 0.001**	4.31	2.42–7.68	** < 0.001**
Unknown	1.98	1.12–3.50	**0.019**	1.76	0.99–3.14	0.056
Bone invasion (yes/no)	1.30	1.00–1.68	**0.047**	–		
GTR (yes/no)	0.92	0.72–1.16	0.458			
RT (yes/no)	1.51	1.19–1.91	**0.001**	–		

Univariate and multivariate Cox regression model were used for survival analysis

*CI* confidence interval, *HR* hazard ratio, *GTR* gross total resection, *RT* radiotherapy

P < 0.05 were marked in bold

The aforementioned results indicated that WHO grade was the most vital factor contributing to worse survival. We thus excluded the patients with unknown tumor grade and only retained 248 patients with definite histological grade for further survival analysis. The result suggested that older age and grade III tumors were significantly associated with higher mortality. Inversely, extent of resection was not associated with survival (supplemental Table S2). In subgroup analysis of histological grade, survival benefit was still not observed for both grade II and III meningiomas (supplemental Fig. S2).

### Validating the impact of surgical resection in an independent dataset

Another 92 contemporary meningioma patients (73 with grade II and 19 with grade III) from SYSUCC were used as a validation dataset. Surgical resection was achieved with Simpson grade I in 40 cases (43.5% of the series), grade II in 34 (36.9%), grade III in 11 (12.0%) and grade IV in 7 (7.6%). Results of Cox regression analysis showed that WHO grade (P = 0.028) was the only independent factors for patients’ OS. Extent of resection was not associated with overall mortality (Table 3). For PFS analysis, histological grade, Ki-67 index and extent of resection were associated with tumor progression. Independent predictors for increased PFS included Simpson grade I, II and III resection (both P < 0.02) and Ki-67 < 5% (P = 0.014; Table 3). Simpson I–III resection was thus defined as GTR in this dataset and patients with GTR had an increased PFS than those with STR (P < 0.001; Fig. 1c). Patients with GTR also displayed a trend to longer OS, but the difference was not significant (P = 0.073; Fig. 1d). These results suggested that GTR did not appear to improve survival, but it was associated with improved PFS.

**Table 3:** Risk factors for progression-free and overall survival on 92 patients with high-grade meningiomas from the Sun Yat-sen University Cancer Center

Variables	Univariate analysis			Multivariate analysis		
	HR	95% CI	P value	HR	95% CI	P value
Factors associated with overall survival						
Year of diagnosis (2008–2015/2000–2007)	4.60	0.54–39.02	0.162	2.73	0.31–24.44	0.361
Gender (male/female)	1.46	0.51–4.17	0.479			
Age (≥ 65 years/< 65 years)	1.36	0.42–4.40	0.609			
KPS (≤ 70/> 70)	1.80	0.56–5.80	0.323			
Tumor location (convex/skull base)	0.40	0.09–1.81	0.236			
Tumor size (> 6 cm/≤ 6 cm)	2.34	0.78–7.02	0.129	3.30	0.95–11.46	0.06
WHO grade (III/II)	6.53	2.04–20.87	**0.002**	5.02	1.12–21.11	**0.028**
Bone invasion (yes/no)	0.91	0.31–2.73	0.869			
Ki-67 (≥ 5%/< 5%)	0.26	0.06–1.16	0.077	0.26	0.05–1.51	0.134
Simpson grade						
IV	1(ref)			1(ref)		
III	0	0–0.01	0.961	0	0–0.01	0.961
II	0.33	0.08–1.34	0.121	0.84	0.16–4.47	0.833
I	0.15	0.03–0.71	0.017	0.45	0.09–2.42	0.355
RT (yes/no)	1.51	0.51–4.42	0.456			
Factors associated with progression-free survival						
Year of diagnosis (2008–2015/2000–2007)	0.95	0.38–2.39	0.920			
Gender (male/female)	1.23	0.61–2.50	0.562			
Age (≥ 65 years/< 65 years)	1.05	0.45–2.45	0.914			
KPS (≤ 70/> 70)	0.96	0.39–2.38	0.937			
Tumor location (convex/skull base)	0.73	0.31–1.70	0.467			
Tumor size (> 6 cm/≤ 6 cm)	1.65	0.76–3.61	0.209			
WHO grade (III/II)	2.36	1.06–5.25	**0.035**	–		
Bone invasion (yes/no)	1.39	0.68–2.83	0.361			
Ki-67 (<5%/≥ 5%)	0.34	0.14–0.84	**0.019**	0.31	0.12–0.80	**0.015**
Simpson grade						
IV	1(ref)			1(ref)		
III	0.21	0.05–0.87	**0.031**	0.16	0.04–0.69	**0.014**
II	0.24	0.09–0.65	**0.005**	0.24	0.09–0.67	**0.006**
I	0.11	0.04–0.33	** < 0.001**	0.11	0.04–0.33	** < 0.001**
RT (yes/no)	1.22	0.58–2.56	0.596			

Univariate and multivariate Cox regression model were used for survival analysis

*CI* confidence interval, *HR* hazard ratio, *KPS* Karnofsky performance status, *RT* radiotherapy

P < 0.05 were marked in bold

### Role of adjuvant radiotherapy on subgroup analysis

Next, we investigate the survival impact of postoperative radiotherapy for the SEER patients. The result of logistic regression suggested that patients with grade III meningiomas are more likely to receive radiotherapy compare with those with grade II tumors (odds ratio [OR] 2.32, 95% CI 1.32–4.08, P = 0.003). It might be the reason why radiotherapy was associated with worse OS in univariate Cox analysis (Table 2). But unexpectedly, significant correlation of resection extent with receipt of radiotherapy was not observed (OR 0.89, 95% CI 0.63–1.26, P = 0.522).

**Fig. 2:**
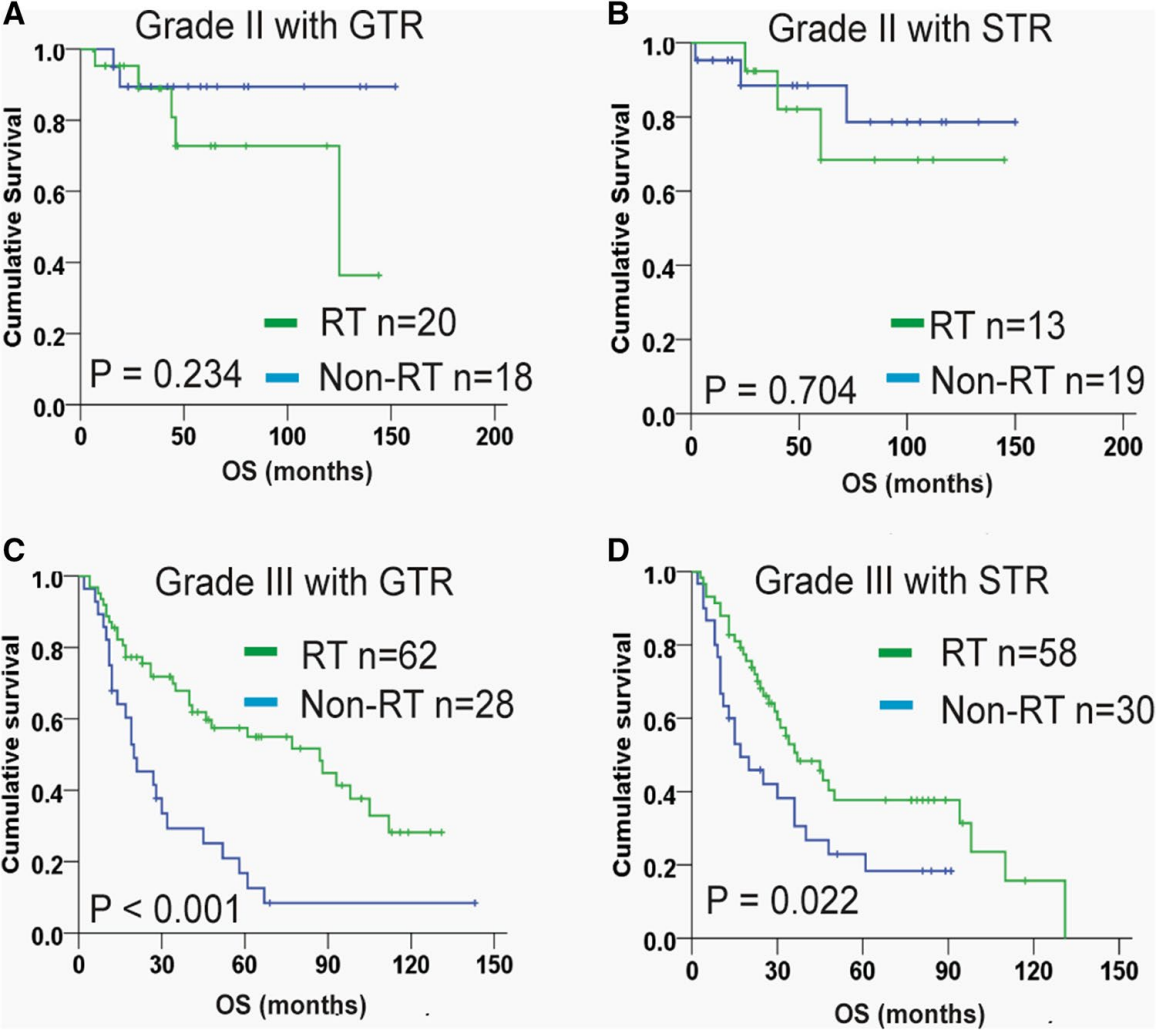
Kaplan–Meier plot for adjuvant radiotherapy on patients in the SEER cohort with grade II meningiomas after GTR (**a**), grade II with meningiomas after STR (**b**); grade III meningiomas after GTR (**c**), and grade III meningiomas after STR (**d**).* GTR* gross total resection,* STR* subtotal resection,* RT* radiotherapy

According to histological grade and extent of resection, 248 patients with grade II or III meningiomas from SEER cohort were divided into four subgroups: grade II with GTR, grade II with STR, grade III with GTR, and grade III with STR (Fig. 2). The Kaplan–Meier OS plots showed that patients with grade II meningiomas received post-surgical radiotherapy displayed similar survival to those who did not received, irrespective of resection extent (both P > 0.05; Fig. 2a, b). For grade III meningiomas, significantly increased OS was observed with adjuvant radiotherapy on both patients undergoing GTR (P < 0.001; Fig. 2c) and STR (P = 0.022; Fig. 2d). In SYSUCC dataset, majority of the cases were grade II meningiomas with complete resection of the tumors (70/92, 76.1%), we thus explored the role of radiotherapy for this population. The results showed that compared with patients who did not received radiotherapy (n = 48), adjuvant radiation (n = 22) was unable to improve OS and PFS of grade II patients with GTR (both P > 0.6; supplemental Figure S3).

## Discussion

SEER program uses ICD-O-3 histology and behavior code to recode the diagnosis and classification of cancer. In the present study, meningiomas identified in SEER database with a behavior code of benign were excluded and the remain patients with malignant or borderline behavior were recognized as high-grade meningiomas for analysis. Although SEER database has been used for reporting the role of extent of resection and radiotherapy for meningiomas before [11–13], this study owns our strength. Firstly, only data between the year 2000 and 2015 were collected and analyzed. The WHO had made major revisions of classification of meningiomas in 2000, followed by minor revisions in 2007 [4], which were accept and supplemented in 2016 criteria [2]. Since histological parameters used for grading assessment in these editions are more standardized compared with previous editions [4], the updated WHO classification has dramatically improved the clinical diagnosis and management of meningiomas. Thus, our results represent the modern management and clinical outcomes of high-grade meningiomas. Secondly, more strict exclusion criteria were applied to improve the representativeness and reliability of the results. It should be noted that many meningiomas recorded in SEER registry are not primary or first tumors. The patients with multiple malignancies history may confound our survival analysis and need to be excluded. Older patients (≥ 80 years) may not suit for standard treatment of brain tumors and was also excluded in this study. Lastly, we have an independent dataset to verify the findings from the SEER cohort.

Meningiomas are typically diagnosed by MRI and CT imaging. Symptomatic patients or tumors larger than 3 cm require treatment. Surgical resection is the first choice to treat meningiomas and obtain tissue samples for pathology [7]. Molecular subtypes have been identified to predict clinical outcome and help treatment decision making for several types of CNS tumors, such as epigenetic subgroups of medulloblastomas and isocitrate dehydrogenase status in diffuse gliomas [2]. However, proposed molecular classification for meningiomas have not entered practice routine. Histological pathology is still the gold standard for diagnosis and grading of these tumors [2, 7]. The vast majority of benign meningiomas can be cured by complete resection of the tumors, whereas, high-grade meningiomas have an increased risk of recurrence, even after GTR [14]. Extent of resection has been reported to affect tumor recurrence [5, 10, 15], but long-term benefit of GTR for survival of patients with high-grade meningiomas remains unclear. Part of the reason is that grade III meningiomas are relatively rare, most survival analysis were based on small sample retrospective researches and results lack high-quality evidence. For another, grade II meningiomas are kinds of borderline malignancies, which possess relatively good survival and need long follow-up duration for analysis. However, diagnostic criteria of meningiomas has underwent numerous modifications since 2000 and led to classification changes on up to 30% of grade II meningiomas [5, 6]. Thus, conclusions from older clinical studies should be treated with caution. Furthermore, even in the modern era of widely application of the adapted WHO criteria, inconsistent results of contemporary studies were observed (see supplemental Table S3) [3, 5, 10, 15–19]. Two studies with large cohorts reported that patients with GTR had significantly increased OS compared with those with STR [17, 18]. On the contrary, results of other retrospective studies showed that extent of resection was independent predictor for PFS, but not for OS [3, 5].

In the present SEER study, with a median follow-up of 144 months on 530 high-grade meningiomas diagnosed between 2000 and 2015, patients with GTR had similar survival to those with STR (Fig. 1a). On subgroups analysis, neither grade II nor grade III patients benefit from GTR. In validation cohort, Simpson grade I–III resection was defined as GTR, which is widely accepted in clinical practice and used for ongoing perspective trials [20, 21]. Extent of resection was not found to be associated with OS, but patients with GTR had significantly improved PFS than those with STR (Fig. 1c). Due to ethics and practice reasons, a randomized trial investigating the survival impact of resection extent for meningiomas is not feasible. This retrospective study with a large cohort provides meaningful data to address this issue. Our results imply that GTR may not improve OS of high-grade meningiomas. Thus, radical surgical strategy that may cause serious complications and even affect patients’ life should be very cautious. Nevertheless, GTR could delay or prevent meningioma progression, indicating the clinical implication of total tumor resection. The concept and implementation of maximal safe resection is reasonable in routine meningioma management.

Besides histological grade and resection extent, as a well-known cellular proliferative marker [22], Ki-67 index was also identified to be associated with tumor progression (Table 3). On the other hand, older age, larger tumor and higher WHO grade demonstrated worse survival (Tables 2 and 3). All these factors need sufficient attention to identify patients at high risk of recurrence and progression, and guide postoperative treatment. High-risk meningiomas should receive radiotherapy after surgery to reduce tumor recurrence. Adjuvant radiation is recommended by the current clinical guideline for grade III meningiomas. However, the level of recommendation is low, because evidences are from results of retrospective series [7, 23, 24]. Patients with incompletely resected grade II meningiomas should also considered radiotherapy, but the benefit lacks consensus [11, 25–27].

A system review concluded that adjuvant radiotherapy significantly improved OS of patients with grade III meningiomas. However, this review was unable to demonstrate a statistically significant benefit for grade II meningiomas [9]. In accordance with the result of this review, we found that significant survival benefit with radiotherapy was only observed for grade III meningiomas, but not for grade II meningiomas regardless of resection extent. More recently, analysis results of the National Cancer Database (a hospital-based cancer registry in the US) showed that adjuvant radiation could confer better survival for incompletely resected grade II meningiomas, but not for complete resected grade II meningiomas [17]. The inconsistent results highlight the need for prospective trials to fully illustrate the impact of radiotherapy for grade II meningiomas. In this study, we also found that postoperative radiation was unable to improve PFS of grade II patients after GTR. Although grade II meningiomas exhibit an increased risk of recurrence compared with benign meningiomas [14], whether radiotherapy can reduce recurrence of completely resected grade II meningiomas remains an major controversy on modern meningioma management [21, 25, 27]. The adjuvant utility of 54 Gy is now being prospectively tested in a randomized trial (versus observation after GTR) for grade II meningiomas in NRG BN003 (NCT03180268). A similar randomized trial (ROAM/EORTC 1308) is also recruiting patients for directly compared radiation with observation in postresection grade II meningiomas [20]. In addition, we should pay attention to the advance in molecular stratifications of meningiomas, which are more correlated to clinical outcomes compared with traditional histological grading. For instance, Sahm et al. [28] developed a DNA methylation-based classification for meningioma survival prediction and Aizer et al. [29] established a prognostic cytogenetic scoring system for guidance of adjuvant treatment.

Several limitations of our study need to be addressed. Besides the retrospective nature, our results are subject to the inherent limits of database review. Some data are not available in the SEER program, such as precise extent of tumor resection in accordance with the Simpson grading scale that is widely used in clinical practice. Although GTR or subtotal resection status are readily recorded, lack of more detailed resection information hinders the development of precise resection standard and surgical guideline. SEER database does not record the status and time of tumor recurrence or progression, which are important information for evaluating tumor control, quality of life and therapeutic effect, especially for borderline cancer like grade II meningiomas. However, these data are collected and analyzed in our independent dataset to provide more comprehensive information to illustrate the effect of surgical resection and radiotherapy on survival of high-grade meningioma.

In conclusion, a large SEER cohort and another independent dataset were used to investigate the survival impacts of extent of resection and adjuvant radiotherapy for high-grade meningiomas treated in the modern era. The results of this analysis provide valuable suggestion for treatment decision making on current routine and indicate the direction for future investigation. Although GTR does not improve OS, it seems to be associated with decreased risk of tumor progression. Post-operative radiotherapy may confer increased OS for grade III meningiomas. For grade II meningiomas, adjuvant radiation is unable to improve OS and PFS for patients after GTR, whereas the role of radiotherapy for incompletely resected patients is still uncertain and needs further validation by prospective clinical trials.

## Electronic supplementary material

Below is the link to the electronic supplementary material.

Electronic supplementary material 1 (DOCX 670 kb)
