# Supplementary material for: Survival impacts of extent of resection and adjuvant radiotherapy for the modern management of high-grade meningiomas
Source: J Neurooncol. 2019 Sep 6;145(1):125–34. doi: 10.1007/s11060-019-03278-w (PMC6775537; doi:10.1007/s11060-019-03278-w)
Supplement: Supplementary file 1 — Electronic supplementary material 1 (DOCX 670 kb) [file 11060_2019_3278_MOESM1_ESM.docx]

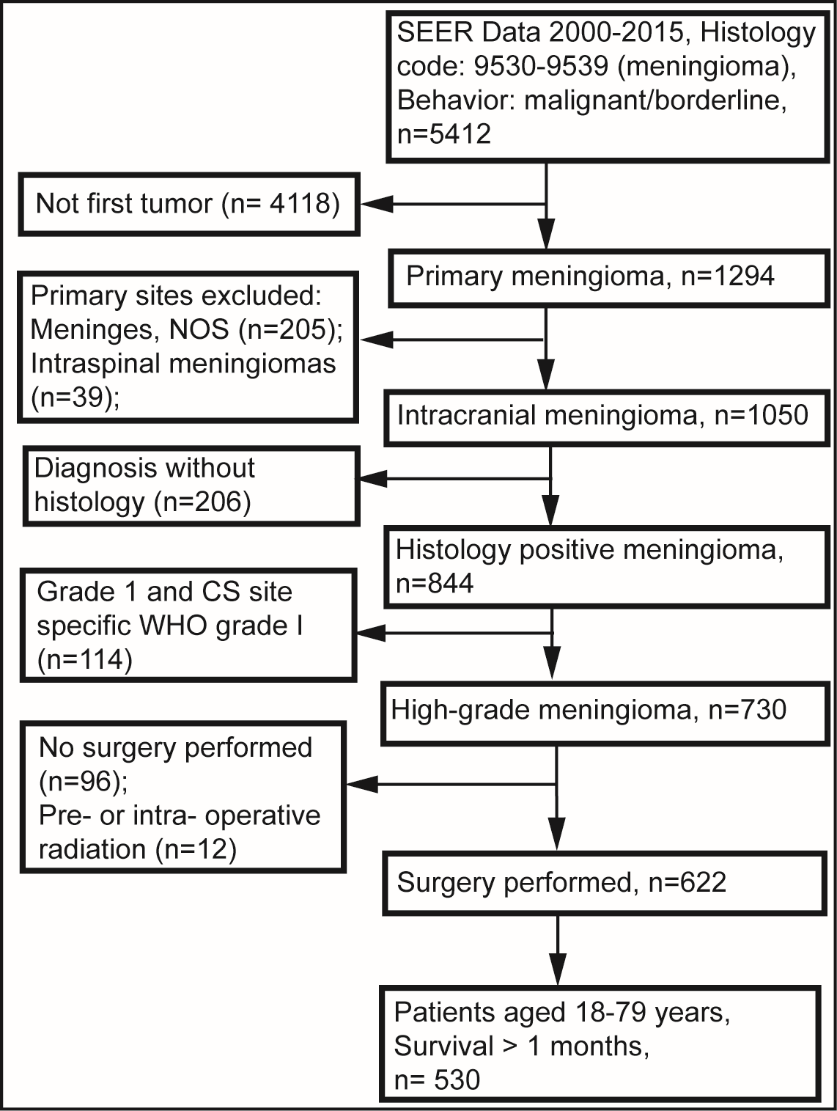


Figure S1 Flow diagram of patient selection in the SEER database


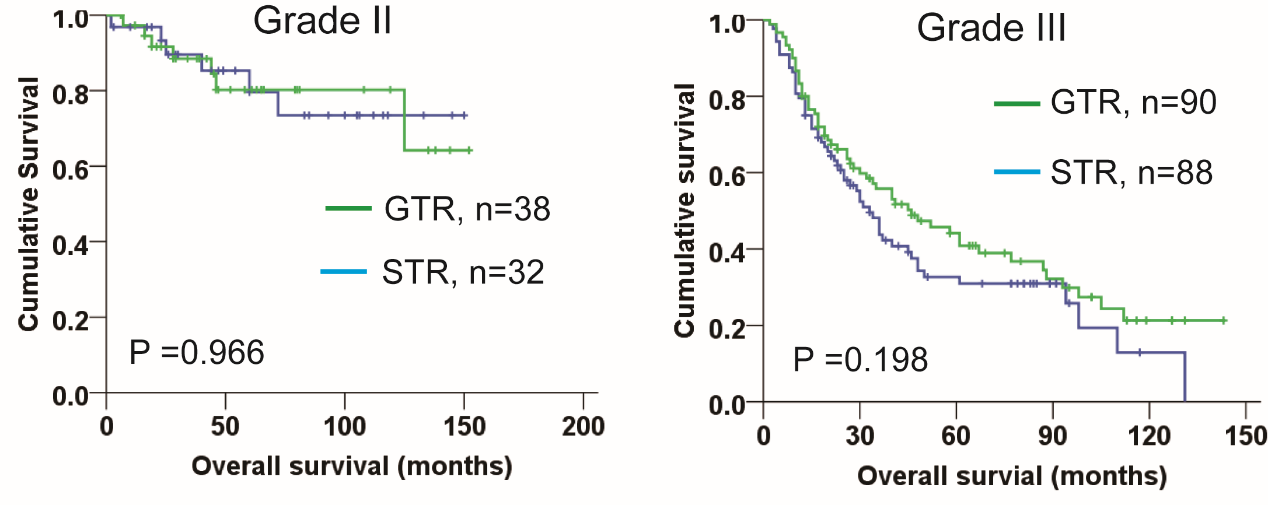


Figure S2 Kaplan-Meier plot for extent of resection on patients with grade II (left) and III (right) meningiomas. GTR, gross total resection; STR, subtotal resection.


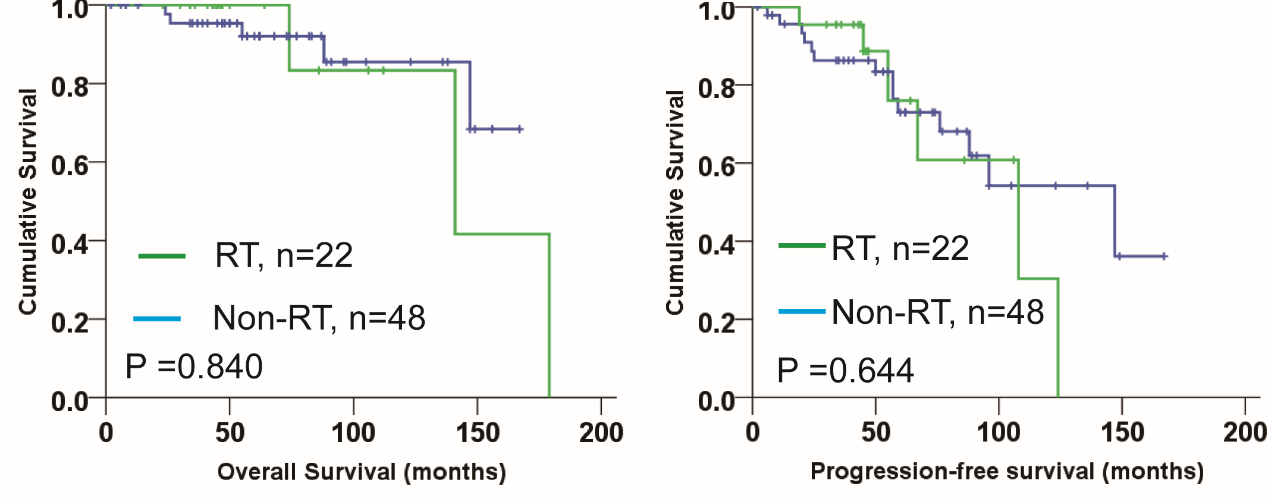


Figure S3 Kaplan-Meier plot for adjuvant radiotherapy on overall survival (left) or progression-free survival (right) of patients with grade II meningiomas after gross total resection. RT, radiotherapy.

**Table S1** Clinical characteristics of patients with high-grade meningiomas from the SEER database

| Variables (n, %) | Total  (n=530) | Total resection  (n=279) | Subtotal resection  (n=251) | P value |
| --- | --- | --- | --- | --- |
| Year of diagnosis |  |  |  |  |
| 2000-2007 | 289 (54.5) | 165 (59.1) | 124 (49.4) | **0.025** |
| 2008-2015 | 241 (45.5) | 114 (40.9) | 127 (50.6) |  |
| Gender |  |  |  |  |
| Male | 244 (46.0) | 128 (45.9) | 116 (46.2) | 0.938 |
| Female | 286 (54.0) | 151 (54.1) | 135 (53.8) |  |
| Age |  |  |  |  |
| <65 | 326 (61.5) | 170 (60.9) | 156 (62.2) | 0.773 |
| ≥65 | 204 (38.5) | 109 (39.1) | 95 (37.8) |  |
| Race |  |  |  |  |
| White | 389 (73.4) | 204 (73.1) | 185 (73.7) | 0.808 |
| Black | 83 (15.7) | 46 (16.5) | 37 (14.7) |  |
| Other | 58 (10.9) | 29 (10.4) | 29 (11.6) |  |
| Tumor location |  |  |  |  |
| Supratentorial | 254 (47.9) | 138 (49.5) | 116 (46.2) | 0.555 |
| Infratentorial | 159 (30.0) | 78 (27.9) | 81 (32.3) |  |
| Unknown | 117 (22.1) | 63 (22.6) | 54 (21.5) |  |
| Laterality |  |  |  |  |
| Left | 179 (33.8) | 95 (34.1) | 84 (33.4) | 0.892 |
| Right | 158 (29.8) | 80 (28.7) | 78 (31.1) |  |
| Bilateral | 32 (6.0) | 16 (5.7) | 16 (6.4) |  |
| Unknown | 161 (30.4) | 88 (31.5) | 73 (29.1) |  |
| Tumor diameter |  |  |  |  |
| ≤6cm | 289 (54.5) | 166 (59.5) | 123 (49.0) | **0.048** |
| >6cm | 86 (16.2) | 42 (15.1) | 44 (17.5) |  |
| Unknown | 155 (29.2) | 71 (25.4) | 84 (33.5) |  |
| Bone invasion |  |  |  |  |
| Yes | 141 (26.6) | 71 (25.4) | 70 (27.9) | 0.526 |
| No | 389 (73.4) | 208 (74.6) | 181 (72.1) |  |
| WHO grade |  |  |  |  |
| II | 70 (13.2) | 38 (13.6) | 32 (12.7) | 0.788 |
| III | 178 (33.6) | 90 (32.3) | 88 (35.1) |  |
| Unknown | 282 (53.2) | 151 (54.1) | 131 (52.2) |  |
| Radiotherapy |  |  |  |  |
| Yes | 233 (44.0) | 119 (42.7) | 114 (45.4) | 0.522 |
| No | 297 (56.0) | 160 (57.3) | 137 (54.6) |  |

Chi-squared test was used for comparison. P<0.05 were in bold.

**Table S1** Clinical characteristics of SEER patients with high-grade meningiomas after propensity score matching

| Variable (n, %) | Total resection  (n=213) | Partial resection  (n=213) | P value |
| --- | --- | --- | --- |
| Gender |  |  |  |
| Male | 97 (45.5) | 99 (46.5) | 0.846 |
| Female | 116 (54.5) | 114 (53.5) |  |
| Age |  |  |  |
| <65 | 135 (63.4) | 131 (61.5) | 0.689 |
| ≥65 | 78 (36.6) | 82 (38.5) |  |
| Race |  |  |  |
| White | 153 (71.8) | 158 (74.2) | 0.801 |
| Black | 37 (17.4) | 32 (15.0) |  |
| Other | 23 (10.8) | 23 (10.8) |  |
| Year of diagnosis |  |  |  |
| 2000-2007 | 112 (52.6) | 107 (50.2) | 0.628 |
| 2008-2015 | 101 (47.4) | 106 (49.8) |  |
| Tumor location |  |  |  |
| Supratentorial | 104 (48.8) | 104 (48.8) | 0.915 |
| Infratentorial | 67 (31.5) | 70 (32.9) |  |
| Unknown | 42 (19.7) | 39 (18.3) |  |
| Laterality |  |  |  |
| Left | 73 (34.3) | 77 (36.2) | 0.909 |
| Right | 65 (30.5) | 62 (29.1) |  |
| Bilateral | 15 (7.0) | 12 (5.6) |  |
| Unknown | 60 (28.2) | 62 (29.1) |  |
| Tumor diameter |  |  |  |
| ≤6cm | 108 (50.7) | 121 (56.8) | 0.408 |
| >6cm | 39 (18.3) | 37 (17.4) |  |
| Unknown | 66 (31.0) | 55 (25.8) |  |
| Bone invasion |  |  |  |
| Yes | 62 (29.1) | 59 (27.7) | 0.747 |
| No | 151 (70.9) | 154 (72.3) |  |
| WHO grade |  |  |  |
| II | 26 (12.2) | 28 (13.1) | 0.934 |
| III | 80 (37.6) | 77 (36.2) |  |
| Unknown | 107 (50.2) | 108 (50.7) |  |
| Radiotherapy |  |  |  |
| Yes | 100 (46.9) | 95 (44.6) | 0.627 |
| No | 113 (53.1) | 118 (55.4) |  |

Chi-squared test was used for comparison.

**Table S2** Risk factors for overall survival on patients with grade II and III meningiomas from the SEER registry (n=248)

| Variables | Univariate analysis | | |  | Multivariate analysis | | |
| --- | --- | --- | --- | --- | --- | --- | --- |
|  | HR | 95% CI | P value |  | HR | 95% CI | P value |
| Year of diagnosis  (2008-2015/ 2000-2007) | 0.92 | 0.60-1.40 | 0.678 |  |  |  |  |
| Gender (male/ female) | 1.13 | 0.80-1.60 | 0.498 |  |  |  |  |
| Age (≥65y/ <65 y) | 2.46 | 1.73-3.51 | **<0.001** |  | 2.51 | 1.76-3.60 | **<0.001** |
| Race |  |  |  |  |  |  |  |
| White | 1(ref) |  |  |  |  |  |  |
| Black | 0.62 | 0.36-1.09 | 0.097 |  | - |  |  |
| Other | 1.03 | 0.62-1.70 | 0.921 |  |  |  |  |
| Tumor location |  |  |  |  |  |  |  |
| Supratentorial | 1(ref) |  |  |  |  |  |  |
| Infratentorial | 1.21 | 0.81-1.80 | 0.356 |  |  |  |  |
| Unknown | 1.24 | 0.77-1.99 | 0.384 |  |  |  |  |
| Laterality |  |  |  |  |  |  |  |
| Left | 1(ref) |  |  |  |  |  |  |
| Right | 0.83 | 0.57-1.20 | 0.318 |  |  |  |  |
| Bilateral | 1.21 | 0.66-2.20 | 0.536 |  |  |  |  |
| Unknown^a^ | - |  |  |  |  |  |  |
| Tumor diameter |  |  |  |  |  |  |  |
| ≤6cm | 1(ref) |  |  |  |  |  |  |
| >6cm | 1.41 | 0.91-2.19 | 0.129 |  | - |  |  |
| Unknown | 1.82 | 1.19-2.80 | **0.006** |  | - |  |  |
| WHO grade (III/II) | 4.91 | 2.76-8.75 | **<0.001** |  | 5.01 | 2.80-8.94 | **<0.001** |
| Bone invasion (yes/no) | 1.27 | 0.86-1.88 | 0.238 |  |  |  |  |
| GTR (yes/no) | 0.84 | 0.59-1.19 | 0.317 |  |  |  |  |
| RT (yes/no) | 0.79 | 0.55-1.13 | 0.201 |  |  |  |  |

Univariate and multivariate Cox regression model were used for survival analysis. P<0.05 were represented in bold. ^a^ n=2. CI: Confidence interval, HR: Hazard ratio, GTR: Gross total resection, RT: Radiotherapy
